# A nontraditional method for reducing thermoelastic stresses of variable thickness rotating discs

**DOI:** 10.1038/s41598-023-39878-w

**Published:** 2023-08-21

**Authors:** A. M. Eldeeb, Y. M. Shabana, T. A. El-Sayed, A. Elsawaf

**Affiliations:** 1 Mechanical Design Department, Faculty of Engineering, Helwan University, P.O. Box 11718, El-Mataria, Cairo, Egypt; 2 Department of Astronautic Science and Mechanics, Harbin Institute of Technology, Harbin, 150001 PR China; 3 Mechanical Engineering Department, College of Engineering and Technology-Cairo Campus, Arab Academy for Science, Technology and Maritime Transport (AASTMT), Cairo, Egypt

**Keywords:** Mechanical engineering, Materials for devices

## Abstract

Stresses reductions and/or raising the load-carrying capacity for a mechanical structure are always great dilemmas for researchers. In this article, a novel method is proposed, and its efficiency is examined for achieving these goals on functionally graded rotating nonuniform thickness discs. The originality of this method relies on comprising a geometrically well-defined area, into the whole structure, with certain homogeneous properties including density, thermal expansion coefficient, and elasticity matrix. This area acts as a reducer of the maximum values of various stress components. The solution of the magnetoelastic/magneto-thermoelastic problem is accomplished using the finite element method. The disc is subjected to partial uniform outer pressure, whereas, upon applying thermal loads; the thermal boundary conditions are considered symmetric. The proposed method is found to be beneficial as the obtained results demonstrated the ability to reduce the maximum stresses with different percentages depending on the location, angular width, and properties of the predefined area. This is reflected by an attainable decrease in the maximum compressive tangential stress and the von Mises stress by approximately 20.7% and 12.5%, respectively, under certain conditions.

## Introduction

Functionally graded materials (FGMs) have gained significant attention in the engineering community for their exceptional properties compared to traditional laminated composites and are widely used in severe working conditions^1,2^. Rotating discs, which are crucial components in many engineering systems, are often made of FGMs. Numerous studies have explored their elastic^3,4^, thermoelastic^5,6^, and elastoplastic static behaviors^7–9^. The dynamic performance of rotating discs has also been investigated in several studies^10–12^. Additionally, the effects of asymmetric loading conditions on their performance has been examined in some research^13,14^.

On the other hand, other scholars considered the magnetoelastic (ME) and magneto-thermoelastic (MTE) behaviors of discs (and their counterparts: cylinders and spheres), which are core items of many applications, such as aerospace industry, supersonic airplanes, rockets/missiles, submarine structures, nuclear energy, electronics, biomedical sector, geophysics, turbines/pumps/compressors rotors, optics, pipes, brake discs, internal combustion engines, ME sensors and actuators^15–18^. For example, Rad and Shariyat^19^ developed a 3D model for FGM discs prone to nonuniform loadings. Additionally, different solution techniques were proposed to obtain the MTE performance of symmetric structures (disc/cylinder/sphere)^20,21^. These methods include for instance the finite element method (FEM) that was used by Zenkour and Abbas^16^ to study the effects of the FGM’s heterogeneity index on the disc’s stresses. Moreover, Zenkour^22^ and Dini et al.^18^ obtained the steady MTE behaviors of multi-layer discs.

In the same vein, different means are sought to alleviate the stresses and/or enhance the sustainability (e.g., loading limits and failure likelihood) of any structure. For example, performing optimization for some parameters is found to be advantageous^23–25^. Also, the concept of grading the material properties along two simultaneous directions has its positive gain^26–28^. In addition, controlling a material’s microstructure, to have a new substance with certain aspired properties^29^ (e.g., yield strength^30^), is also beneficial. The latter seems to be complicated; however, with the grand advancement in technology and fabrication techniques, it became attainable and easily accomplished. For instance, two methods are being used for this sake: modified slip-casting method^31^ and fused deposition modeling method^32^.

In light of the extensive literature, efforts are being exerted on exploring methods to mitigate the stress and increase the loading capacity of rotating discs. Thus, this article proposes a novel and atypical approach to material customization under asymmetric loading conditions. This unconventional method involves incorporating a predefined area within the disc’s FGM domain that has constant values for selected properties. The primary objective of this novel area is to reduce induced stress levels. The article explores through the FEM the effects of this area, including its properties, angular width, and position, on the disc’s MT and MTE behaviors. The judgment on the success or failure of the proposed idea will be evaluated based on the reduction of the maximum stress components including the von Mises stress, to avoid plasticity occurrence.

## Problem formulation

**Figure 1:**
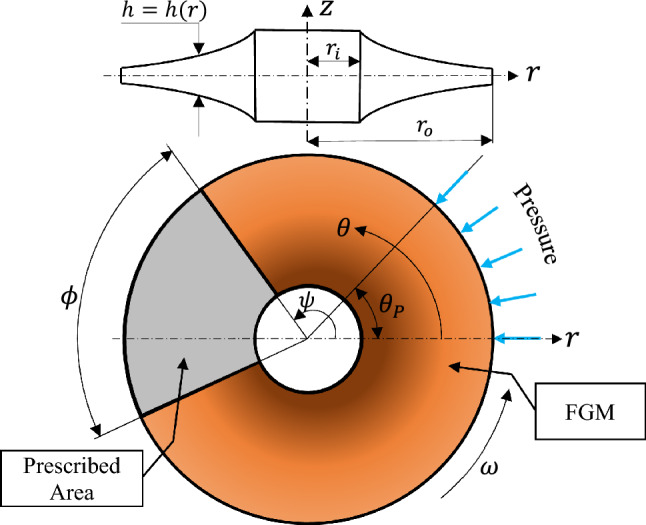
Schematic drawing of the proposed rotating annular variable thickness disc. The gradient-filled brown area represents the FGM, while the gray area, with an angular width of $\phi$, is made of homogeneous material with constant selected properties. The outer pressure angle is denoted by ${\theta }_{P}$.

In polar coordinates ($r\theta z$), Fig. 1 depicts a disc with inner and outer radii $r_{i}$ and $r_{o}$, respectively. The disc has a nonuniform thickness $h = h\left( r \right)$, and rotates with angular speed $\omega$. It is comprised of two portions with distinct properties. One portion is formed of FGM (i.e., $\psi > \theta > \psi + \phi$), while the other, named as the prescribed area ($\psi \le \theta \le \psi + \phi$), is made of a material with selected constant properties. The angles $\psi$ and $\phi$ will be defined in the following section, and $\theta_{P}$ is the angle of the applied outer pressure.

The thermomechanical equilibrium equations are written as^33^:

1 $$ \frac{1}{rh}\left( {rhk_{r} T_{,r} } \right)_{,r} + \frac{1}{{r^{2} h}}\left( {hk_{\theta } T_{,\theta } } \right)_{,\theta } = 0 $$

2 $$ \left( {rh\sigma_{rr} } \right)_{,r} + \left( {h\sigma_{r\theta } } \right)_{,\theta } - h\sigma_{\theta \theta } + F_{r} + rhf_{r} = 0 $$

3 $$ \left( {rh\sigma_{r\theta } } \right)_{,r} + \left( {h\sigma_{\theta \theta } } \right)_{,\theta } + h\sigma_{r\theta } + F_{\theta } + rhf_{\theta } = 0 $$

where the comma denotes partial differentiation, $T$ is the temperature, and $$ {\mathbf{k}} = \left[ {\begin{array}{*{20}c} {k_{r} } & 0 \\ 0 & {k_{\theta } } \\ \end{array} } \right]$$ is the thermal conductivity. $F_{r} = \rho h\omega^{2} r^{2}$ is the centrifugal force, and $F_{\theta } = \rho h\omega_{,t} r^{2}$ is the tangential force that exists when $\omega_{,t} \ne 0$, where $t$ is the time variable, and $\rho$ represents the material’s density. In terms of $h$, it is proposed to change with the radial position $r$ through the following relation^34^:

4 $$ h\left( r \right) = H\left( {1 - {\mathcalligra{n}}_{1} \left( {\frac{r}{{r_{o} }}} \right)} \right)^{{\mathcalligra{n}}_{2} } $$

with $H$ is an imaginary thickness at $r = 0$, and ${\mathcalligra{n}}_{j}$ ($j = 1, 2$) is a geometrical parameter.

Furthermore, since the disc has a large diameter-to-thickness ratio; the state of plane stress becomes a valid hypothesis; hence, the constitutive relation is^35^:

5 $$ {{\varvec{\upsigma}}} = {\mathbf{C{{\varvec{\upvarepsilon}}}}} - {{\varvec{\uplambda}}}T $$

where ${{\varvec{\upsigma}}}$ is the stress field of three components: $\sigma_{rr}$, $\sigma_{\theta \theta }$, and $\sigma_{r\theta }$ that are the radial, tangential, and shear stresses, respectively. Also, ${\mathbf{C}}$ is the elasticity matrix with the following non-zero components: $C_{11} = \frac{E}{{1 - v^{2} }}$, $C_{12} = \frac{Ev}{{1 - v^{2} }}$ and $C_{66} = \frac{{\left( {C_{11} - C_{12} } \right)}}{2}$, where $E$ and $v$ resemble the material’s elastic modulus and Poisson’s ratio, respectively. Besides, ${{\varvec{\upvarepsilon}}}$ refers to the strain components given through the kinematics relation (Eq. (6)), with $u$ and $\vartheta$ are the radial and tangential displacements, in turns^34^. In addition, ${{\varvec{\uplambda}}}$ (stress-temperature coefficient vector) is calculated through Eq. (7) with ${{\varvec{\upalpha}}}$ is the thermal expansion coefficient vector^36^.

6 $$ {{\varvec{\upvarepsilon}}} = \left\{ {\begin{array}{*{20}c} {\varepsilon_{rr} } \\ {\varepsilon_{\theta \theta } } \\ {2\varepsilon_{r\theta } } \\ \end{array} } \right\} = \left\{ {\begin{array}{*{20}c} {u_{,r} } \\ {\left( {u + \vartheta_{,\theta } } \right)/r} \\ {\vartheta_{,r} + \left( {u_{,\theta } - \vartheta } \right)/r} \\ \end{array} } \right\} $$

7 $$ \begin{array}{*{20}c} {{{\varvec{\uplambda}}} = {\mathbf{C\alpha }} \to \left\{ {\begin{array}{*{20}c} {\lambda_{1} } \\ {\lambda_{2} } \\ {\lambda_{3} } \\ {\lambda_{6} } \\ \end{array} } \right\} = \left[ {\begin{array}{*{20}c} {C_{11} } & {C_{12} } & {C_{12} } & 0 \\ {} & {C_{11} } & {C_{12} } & 0 \\ {} & {} & {C_{11} } & 0 \\ {sym} & {} & {} & {C_{66} } \\ \end{array} } \right]\left\{ {\begin{array}{*{20}c} {\alpha_{1} } \\ {\alpha_{2} } \\ {\alpha_{3} } \\ 0 \\ \end{array} } \right\} } \\ \end{array} $$

Moreover, in Eqs. (2) and (3), the two components of ${f}_{j}$ ($j=r$,$\theta$) are related to Lorentz force arising due to the presence of the constant and axial magnetic field (${\mathcal{H}}_{z}$), and are given as^37^:

8 $$ \begin{array}{*{20}c} {f_{r} = \mu {\mathcal{H}}_{z}^{2} \left( {u_{,r} + \frac{u}{r} + \frac{1}{r}\vartheta_{,\theta } } \right)_{,r} ,} & {f_{\theta } = \frac{{\mu {\mathcal{H}}_{z}^{2} }}{r}\left( {u_{,r} + \frac{u}{r} + \frac{1}{r}\vartheta_{,\theta } } \right)_{,\theta } } \\ \end{array} $$

where $\mu ={\mu }_{0}\eta$ is the material’s magnetic permeability with ${\mu }_{0}=4\pi \times {10}^{-7}\mathrm{H}/\mathrm{m}$ is the permeability of space, and $\eta$ is the material’s relative magnetic permeability^18^.

## Material gradation

In the current analyses, the disc is made of two parts with dissimilar material compositions. The first part is made of FGMs (the gradient-filled brown area shown in Fig. 1). The material properties are radially graded as follows^38^:

9 $$ \beta \left( {r,\psi > \theta > \psi + \phi } \right) = \beta_{m} + \left( {\beta_{c} - \beta_{m} } \right)\left( {\frac{{r - r_{i} }}{{r_{o} - r_{i} }}} \right)^{j} $$

where $\beta$ denotes a generic material property, and $j$ refers to the heterogeneity index. Also, the two subscripts $m$ and $c$ stand for metal and ceramic, respectively.

Conversely, the other part, or area, of the disc, which has an angular width of $\phi$ and named as the prescribed area (shaded in gray in Fig. 1), is located at angle $\psi$. This area is assumed to be perfectly bonded to the FGM area. It is introduced to be examined as being effective as an unconventional method in reducing the disc’s induced stresses. In addition, it has special material characteristics compared to the FGM area. It has specific and constant selected properties (${\beta }^{*}$). The rest of the properties, other than the selected ones, would follow Eq. (9). In other words, this area has:

10 $$ \beta^{*} \left( {r,\psi \le \theta \le \psi + \phi } \right) = x_{{\beta^{*} }} \beta_{{\xi_{{\beta^{*} }} }} \;\;\;\;(\xi_{{\beta^{*} }} = m\;{\text{or}}\;c) $$

where $x$ is named as the property fraction, which is a positive number ($0.25$, $0.5$ or $1$), and ${\xi }_{{\beta }^{*}}$ is a symbol standing for either $m$ or $c$. For simplicity, ${\beta }^{*}$ would include either of $\rho$, $\alpha$ and $\mathbf{C}$. In practical applications, this idea can be achieved through controlling the microstructure of the materials that can be conducted by the material’s processing approach with the aid of the great advancement and technology available globally.

## Finite element formulation

A finite element (FE) algorithm is built by the authors through the MATLAB software to solve for $${\mathbf{X}} = \left\{ {\begin{array}{*{20}c} {\mathbf{U}} & {\mathbf{T}} \\ \end{array} } \right\}$$, where $${\mathbf{U}} = \left\{ {\begin{array}{*{20}c} u & \vartheta \\ \end{array} } \right\}$$. In FEM, ${\mathbf{X}}$ is connected to its nodal values through the shape functions ($N$)^39,40^:

11 $$ {\mathbf{X}} \approx \mathop \sum \limits_{i = 1}^{{n_{n} }} N_{i}^{e} {\mathbf{X}}_{i} $$

where ${n}_{n}$ is the number of nodes per element, and ${N}_{i}^{e}$ is read as: $N$ at the $i$th node of the element $e$.

Thereafter, the FE symbolic equation (${\mathbf{KX}} = {\mathbf{R}}$) is derived using the standard Galerkin’s procedures^39^. In that equation, ${\mathbf{K}}$ represents the global stiffness matrix, and ${\mathbf{R}}$ denotes the external force vector. That symbolic equation is expanded as below^39^:

12 $$ \mathop \sum \limits_{e = 1}^{{n_{e} }} \left( {\left[ {\begin{array}{*{20}c} {{\mathbf{K}}_{{{\mathbf{UU}}}}^{{\varvec{e}}} } & {{\mathbf{K}}_{{{\mathbf{UT}}}}^{{\varvec{e}}} } \\ 0 & {{\mathbf{K}}_{{{\mathbf{TT}}}}^{{\varvec{e}}} } \\ \end{array} } \right]\left\{ {\begin{array}{*{20}c} {{\mathbf{U}}^{{\varvec{e}}} } \\ {{\mathbf{T}}^{{\varvec{e}}} } \\ \end{array} } \right\} = \left\{ {\begin{array}{*{20}c} {{\mathbf{R}}_{{\mathbf{U}}}^{{\varvec{e}}} } \\ 0 \\ \end{array} } \right\}} \right) $$

with ${n}_{e}$ is the total number of elements. Each term in Eq. (12) is calculated as follows:

13 $$ \left\{ {\begin{array}{*{20}l} {{\mathbf{K}}_{{{\mathbf{UU}}}}^{{\varvec{e}}} = \mathop \smallint \limits_{{\Omega_{e} }} rh\left[ {{\mathbf{B}}_{{\mathbf{U}}} } \right]\left[ {\mathbf{C}} \right]\left[ {{\mathbf{B}}_{{\mathbf{U}}} } \right]{\text{d}}\Omega_{e} - \mathop \smallint \limits_{{{\Omega }_{e} }} rh\mu {\mathcal{H}}_{z}^{2} \left[ {{\hat{\mathbf{N}}}} \right]\left[ {{\mathbf{B}}_{{{\varvec{\upmu}}}} } \right]\left[ {\varvec{f}} \right]d\Omega_{e} } \hfill \\ {{\mathbf{K}}_{{{\mathbf{UT}}}}^{{\varvec{e}}} = - \mathop \smallint \limits_{{\Omega_{e} }} rh\left[ {{\mathbf{B}}_{{\mathbf{U}}} } \right]\left[ {{\varvec{\uplambda}}} \right]\left[ {\mathbf{N}} \right]{\text{d}}\Omega_{e} } \hfill \\ {{\mathbf{K}}_{{{\mathbf{TT}}}}^{{\varvec{e}}} = \mathop \smallint \limits_{{\Omega_{e} }} rh\left[ {{\mathbf{B}}_{{\mathbf{T}}} } \right]\left[ {\mathbf{k}} \right]\left[ {{\mathbf{B}}_{{\mathbf{T}}} } \right]{\text{d}}\Omega_{e} } \hfill \\ {{\mathbf{R}}_{{\mathbf{U}}}^{{\varvec{e}}} = \mathop \smallint \limits_{\Gamma } rh\left[ {\mathbf{N}} \right]\sigma_{n} d\Gamma + \mathop \smallint \limits_{{\Omega_{e} }} \left[ {\mathbf{N}} \right]\left[ {\mathbf{F}} \right]{\text{d}}\Omega_{e} } \hfill \\ \end{array} } \right. $$

where $$\left[ {{\hat{\mathbf{N}}}} \right] = \left[ {\begin{array}{*{20}c} {\left[ {\mathbf{N}} \right]} & {\left[ {\mathbf{N}} \right]} \\ {\left[ {\mathbf{N}} \right]} & {\left[ {\mathbf{N}} \right]} \\ \end{array} } \right]_{{2n_{n} \times 2}}$$, $${\mathbf{F}} = \left\{ {\begin{array}{*{20}c} {F_{r} } \\ {F_{\theta } } \\ \end{array} } \right\}$$, and $${\varvec{f}} = \left\{ {\begin{array}{*{20}c} {f_{r} } \\ {f_{\theta } } \\ \end{array} } \right\}$$.

The integrations of Eq. (13) are accomplished through the gauss quadrature method with nine gauss points within each element that has an area of ${\Omega }_{e}$, such that $\mathrm{d}{\Omega }_{\mathrm{e}}=\mathrm{drd\theta }$. In addition, ${{\varvec{\upsigma}}}_{n}$ identifies the traction on a certain part $\Gamma$ of the boundary^39,41^. Furthermore, ${\mathbf{B}}_{\mathbf{U}}$ represents the strain–displacement matrix, ${\mathbf{B}}_{\mathbf{T}}$ is the gradient matrix (see for instance Refs^39,42^. for their definitions.), and ${\mathbf{B}}_{{\varvec{\upmu}}}$ is related to the magnetic field and is determined as follows:

14 $$ {\mathbf{B}}_{{{\varvec{\upmu}}}} = \left[ {\begin{array}{*{20}c} {\left[ {\mathbf{N}} \right]_{,rr} + \left[ {\mathbf{N}} \right]_{,r} r^{ - 1} - \left[ {\mathbf{N}} \right]r^{ - 2} } & {\left[ {\mathbf{N}} \right]_{,r\theta } r^{ - 1} - \left[ {\mathbf{N}} \right]_{,\theta } r^{ - 2} } \\ {\left[ {\mathbf{N}} \right]_{,r\theta } r^{ - 1} + \left[ {\mathbf{N}} \right]_{,\theta } r^{ - 2} } & {\left[ {\mathbf{N}} \right]_{,\theta \theta } r^{ - 2} } \\ \end{array} } \right]_{{2 \times 2n_{n} }} $$

Finally, $\mathbf{X}$ can be easily obtained using matrix calculus. Then, ${\varvec{\upvarepsilon}}$ and ${\varvec{\upsigma}}$ are computed directly after applying the proper boundary conditions^41^.

## Algorithm verification

In this section, the validity of the proposed FEM formulation is examined through regenerating the results of three examples that exist in previous literature.

**Figure 2:**
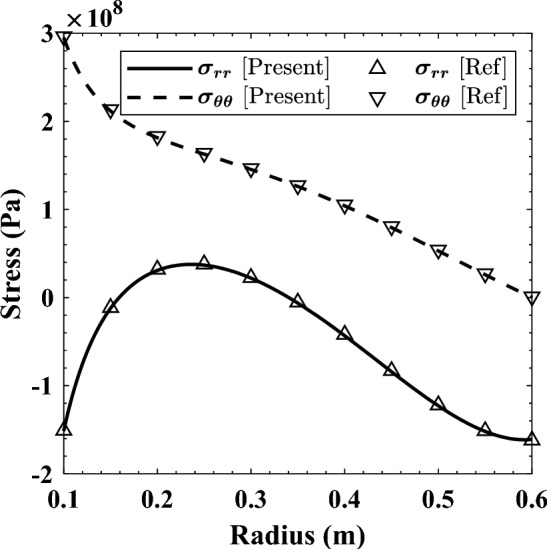
Comparison between the stresses’ distributions obtained through FEM and in Ref ^43^. at $\theta =0^\circ$.

The first example is a nonuniform thickness clamped-free disc studied using the Runge–Kutta’s method by Hassani et al.^43^. It rotates with $\omega =300 \mathrm{rad}/\mathrm{s}$, and is prone to a temperature field defined by Eq. (15). Geometrically, the disc’s thickness is defined via the power-law given in Eq. (16) with ${r}_{i}=0.1 \mathrm{m}$ and ${r}_{o}=0.6 \mathrm{m}$. Table 1 in Ref ^43^. lists the numerical values of the material properties that were graded according to Eq. (9) with $j=2$. Since this problem is completely symmetric (${\sigma }_{r\theta }=0$); there is no need to investigate the $360^\circ$ model of the disc. Only its quarter is modeled with proper symmetric boundary conditions (BCs) at $\theta =0^\circ$ and $90^\circ$^41^. Figure 2 shows the distribution of stresses obtained by the current FEM solution and the method used by Hassani et al.^43^.

15 $$ T\left( r \right) = 100 + \left( {300 - 100} \right)\left( {\frac{{r - r_{i} }}{{r_{o} - r_{i} }}} \right)^{2} \left( {^\circ {\text{C}}} \right) $$

16 $$ h\left( r \right) = 0.1\left( {r/r_{o} } \right)^{0.5} \left( {\text{m}} \right) $$

**Table 1:** Mechanical properties and boundary conditions of FGM cylinder^38^.

Mechanical property^†^		Boundary conditions	
Elastic modulus ($\mathrm{GPa}$)	Poisson’s ratio		
${E}_{i}=200$	${v}_{i}=0.28$	${\sigma }_{rr}\left({r}_{i},\theta \right)={P}_{0}\mathrm{cos}\left(2\theta \right)$	${\sigma }_{rr}\left({r}_{o},\theta \right)=0$
${E}_{o}={E}_{i}/3$	${v}_{o}=0.28$	${\sigma }_{r\theta }\left({r}_{i},\theta \right)=0$	${\sigma }_{r\theta }\left({r}_{o},\theta \right)=0$

† Eq. (9) is used to describe the variation of $E$ and $v$ with ${j}=1$.

The second example is presented to verify the solution ability in obtaining the ME behaviors of a nonrotating cylinder with ${r}_{i}=0.2 {r}_{o}$. It was subjected to uniform magnetic field (${\mathcal{H}}_{z}=2.23\times {10}^{9}\mathrm{ A}/\mathrm{m}$), with the magnetic permeability varying as $\mu ={\mu }_{0}\times {r}^{-2}$^21^. The outer surface was free-of-stress, while at $r={r}_{i}$ a uniform and symmetric pressure with a value of ${P}_{0}$ existed; thus, symmetric BCs prevails. Also, the following quantities were used: $\frac{E\left(1-v\right)}{\left(1+v\right)\left(1-2v\right)}=47.3 \mathrm{GPa}$, and $\frac{Ev}{\left(1+v\right)\left(1-2v\right)}=28.8 \mathrm{GPa}$^21^; so that, after some mathematical manipulations, it yields:

17 $$ E = 25.5 \times r^{ - 2} \;{\text{GPa}}, v = 0.378 $$

However, in this example the plane strain conditions were used; therefore, this conversion is conducted to Eq. (5)^44^:

18 $$ E = E/\left( {1 - v^{2} } \right), v = v/\left( {1 - v} \right) $$

**Figure 3:**
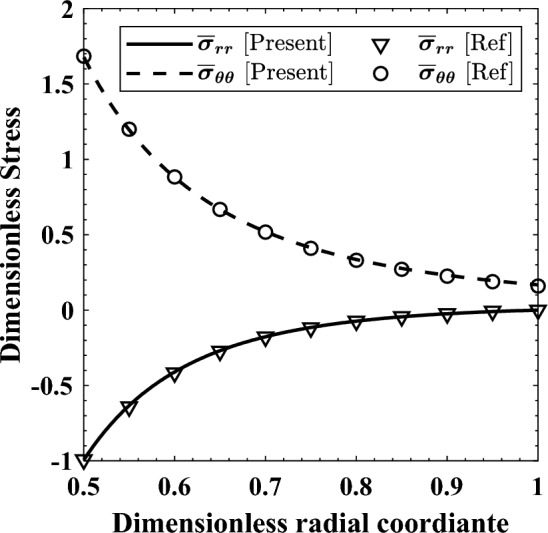
Dimensionless stresses distributions for a cylinder subjected to magnetic field.

Results are presented in a dimensionless form according to Eq. (19), which is used henceforth, and are depicted in Fig. 3.

19 $$ \overline{\sigma }_{ij} = \sigma_{ij} /P_{0} ,\;(i,\;j = r,\;\theta ) $$

**Figure 4:**
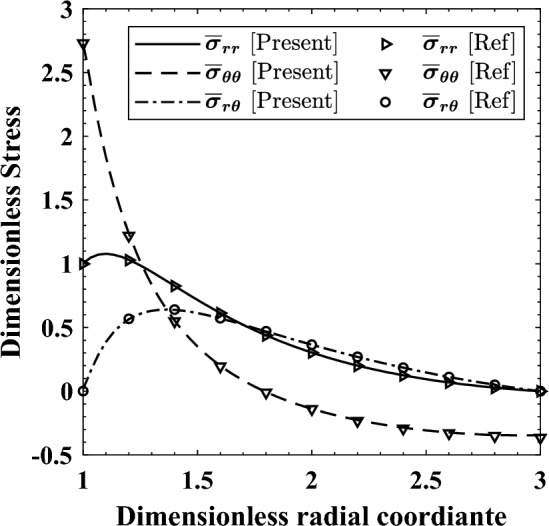
Comparison between the radial variation of ${\bar{\sigma }}_{rr}$, ${\bar{\sigma }}_{\theta \theta }$, and ${\bar{\sigma }}_{r\theta }$ calculated through the current FE algorithm and in Ref.^38^. ${\bar{\sigma }}_{rr}$ and ${\bar{\sigma }}_{\theta \theta }$ are drawn at $\theta =0^\circ$, and ${\bar{\sigma }}_{r\theta }$ is calculated at $\theta =45^\circ$.

The third and final example is a nonrotating cylinder (plane strain) with ${r}_{o}=3{r}_{i}$ studied by Li and Liu^38^. Table 1 lists the numeric values of the mechanical properties, and the associated BCs with ${P}_{0}=100 \mathrm{MPa}$. This example is re-examined to benchmark the capability of solving models with asymmetric BCs. Figure 4 portrays the resulting dimensionless stresses.

It is observed from the previous three verification examples that the obtained outcomes using the present model are in coherence with the results published in the literature. Therefore, the current solution scheme is robust and can be used for predicting the behaviors of discs with different loads and various boundary conditions.

## Results and discussion

As intended in this work, mitigation of the induced ME and MTE stresses of a nonuniform thickness FGM disc is sought. This is accomplished through fixing one property or more within the prescribed area of the disc’s domain. On the other hand, the disc has inner and outer radii of $0.2 \mathrm{m}$ and $0.5 \mathrm{m}$, respectively. The parameters of Eq. (4) are: $H=0.1 \mathrm{m}$, $\mathcalligra{n}_{1}=0.415196$, and $\mathcalligra{n}_{2}=3$^8^. It rotates with $\omega =700\mathrm{ rad}/\mathrm{s}$ (${\omega }_{,t}=0$; thus ${F}_{\theta }=0$), and is subjected to an axial magnetic field of ${\mathcal{H}}_{z}={10}^{6} \mathrm{A}/\mathrm{m} .$

Table 2 lists the material properties assigned in this work. Equation (9) labels the FGM gradation model (${j}=1$), and Eq. (10) describes the selected properties variation within the predefined area. Also, $\phi$ is set to $45^\circ$, and $\psi$ has a value of $0^\circ$, $90^\circ$, or $180^\circ$.

**Table 2:** Material properties of the FGM constituents^16,18^.

	$E$ $\left(\mathrm{GPa}\right)$	$\rho$ $\left(\mathrm{kg}/{\mathrm{m}}^{3}\right)$	$k$^‡^ $\left(\mathrm{W}/\mathrm{mK}\right)$	$\eta$	$v$	$\alpha$^†^ $\left({10}^{-6}/^\circ{\rm C} \right)$
Metal (Aluminum)	$70$	$2700$	$233$	$2.3$	$0.35$	$23.4$
Ceramic (Zirconia)	$151$	$5700$	$2.09$	$1$	$0.3$	$10$

^‡^$${k}_{r}={k}_{\theta }=k$$.

^†^$${\alpha }_{1}={\alpha }_{2}={\alpha }_{3}=\alpha $$.

Concerning the FE scheme, the eight-node (${n}_{n}=8$) isoparametric 2D element is used to discretize the disc’s domain. It is found that convergence occurs at ${n}_{e}=20000$ elements after examining different numbers of elements.

### Case (1): Magnetoelastic (ME) loading

**Figure 5:**
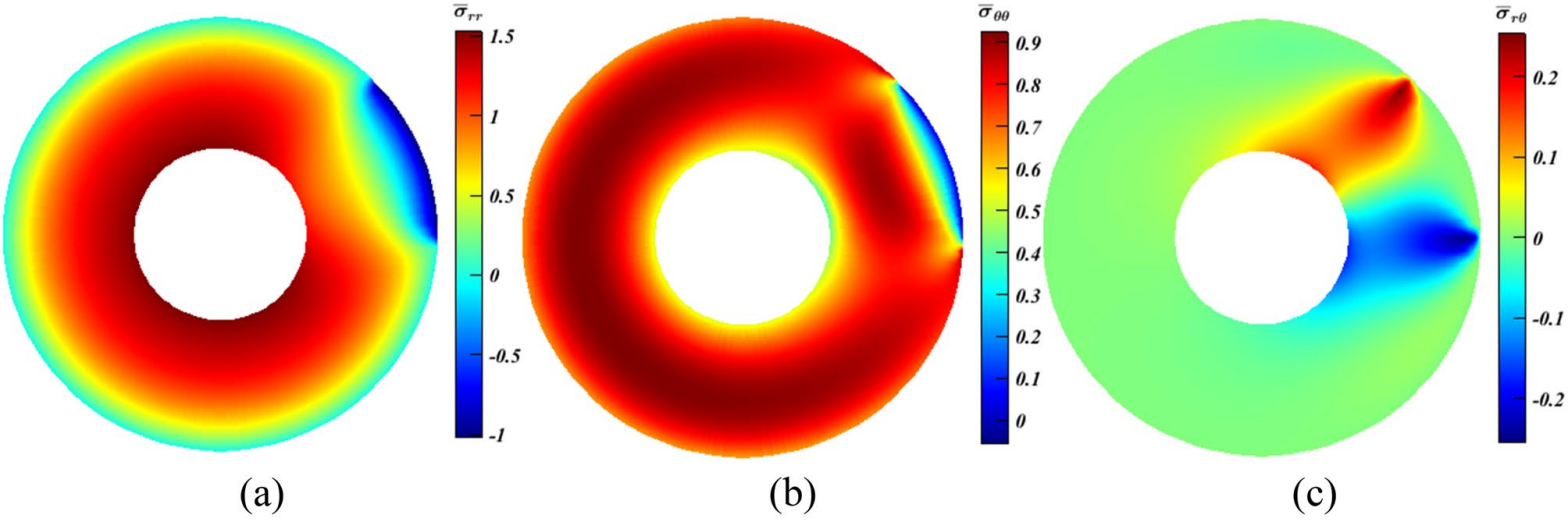
Dimensionless stress contours for a rotating disc under partial normal pressure. (**a**) ${\bar{\sigma }}_{rr}$, (**b**) ${\bar{\sigma }}_{\theta \theta }$, and (**c**) ${\bar{\sigma }}_{r\theta }$.

In the beginning, the analysis commences with a disc having a fixed inner surface ($u\left({r}_{i},\theta \right)=\vartheta \left({r}_{i},\theta \right)=0$), and is subjected to partial uniform normal loading at the outer surface; such that ${\sigma }_{rr}\left({r}_{o},0\le \theta \le {\theta }_{P}\right)=-{P}_{0}$, where ${P}_{0}=100\mathrm{MPa}$ and ${\theta }_{P}=45^\circ$ (see Fig. 1 for its description). For the sake of comparison, Fig. 5 depicts the ME response using Eq. (9) only.

It is observed from Fig. 5a that ${\bar{\sigma }}_{{rr}_{max}}$ and ${\bar{\sigma }}_{{rr}_{min}}$ stood at $\sim 1.5$ and $-1$, respectively. The critical tensile ${\bar{\sigma }}_{\theta \theta }$ is found to be almost $0.92$ (Fig. 5b), while the maximum compressive value is negligible (henceforth $max$ and $min$ denote the greatest values of tensile and compressive stresses in turns). Also, due to the lack of shear traction on any surface; the upper and lower limits of ${\bar{\sigma }}_{r\theta }$ (Fig. 5c) have similar value of approximately $\pm \hspace{0.17em}0.27$. Therefore, it can be concluded that the role of ${\bar{\sigma }}_{r{r}_{min}}$, ${\bar{\sigma }}_{\theta {\theta }_{min}}$ and ${\bar{\sigma }}_{r\theta }$ is negligible compared to ${\bar{\sigma }}_{r{r}_{max}}$ and ${\bar{\sigma }}_{\theta {\theta }_{max}}$.

In order to decrease the values of the extreme stresses, Eq. (10) is used along with Eq. (9) yielding an area with certain homogeneous material properties. Table 3 lists the ME stress values at different values of $\psi$, and ${x}_{{\beta }^{*}}$ while ${\beta }^{*}$ included $\rho$ only.

**Table 3:** Stress values obtaibed at different ${\varvec{\psi}}$ by varying ${\varvec{\rho}}$ only according to Eq. (10).

Stress	Reference value	Equation (10)		$\psi$		
		${x}_{\rho }$	${\xi }_{\rho }$	$180^\circ$	$90^\circ$	$0^\circ$
${\bar{\sigma }}_{{rr}_{max}}$	$1.525$	$1$	$m$	$1.538$	$1.528$	$1.538$
			$c$	$1.966$	$1.986$	$1.563$
		$0.5$	$m$	$1.548$	$1.538$	$1.548$
			$c$	$1.537$	$1.528$	$1.537$
${\bar{\sigma }}_{{\theta \theta }_{max}}$	$0.924$	$1$	$m$	$0.884$	$0.914$	$1.011$
			$c$	$0.988$	$0.973$	$0.944$
		$0.5$	$m$	$0.876$	$0.935$	$1.074$
			$c$	$0.885$	$0.912$	$1.004$

It is seen that the variation of $\psi$ and ${x}_{\rho }$ led to an increase in the values of ${\bar{\sigma }}_{{rr}_{max}}$ due to change in ${F}_{r}$. For example, this growth reached $2.5\%$ at $\psi =0^\circ$, ${x}_{\rho }=1$ and ${\xi }_{\rho }=c$. Alternatively, at $\psi =90^\circ$, ${x}_{\rho }=0.5$ and ${\xi }_{\rho }=c$, a marginal increase is detected ($<0.2\%$). In terms of ${\bar{\sigma }}_{{\theta \theta }_{max}}$, a swing in behavior can be witnessed. At ${x}_{\rho }=0.5$ or 1, and ${\xi }_{\rho }=m$, ${\bar{\sigma }}_{{\theta \theta }_{max}}$ dropped by around $5\%$ at $\psi =180^\circ$, while it increased to $1.074$ at $\psi =0^\circ$ with ${x}_{\rho }=0.5$ and ${\xi }_{\rho }=m$. Also, using $\psi =0^\circ$ would yield larger stresses compared to $\psi =90^\circ$ and $180^\circ$ under this state of loading. Additionally, it is noticed that using ${x}_{\rho }=0.5$ with ${\xi }_{\rho }=c$ would yield nearly analogous results to the case of using ${\xi }_{\rho }=m$ at ${x}_{\rho }=1$.

Afterwards, seeking further reductions in the stresses and/or raising the load-carrying capacity; $\mathbf{C}$ is added to $\rho$ to follow Eq. (10). Table 4 illustrates the stresses values at different values of $\psi$ and ${\xi }_{{\beta }^{*}}$.

**Table 4:** Stress values obtained at different ${\varvec{\psi}}$ by varying ${\varvec{\rho}}$ and $\mathbf{C}$ according to Eq. (10).

Stress	Reference value	Equation (10)		$\psi$		
		${x}_{\rho }={x}_{\mathbf{C}}$	${\xi }_{{\beta }^{*}}$	$180^\circ$	$90^\circ$	$0^\circ$
${\bar{\sigma }}_{{rr}_{max}}$	1.525	$1$	$m$	1.541	1.531	1.544
			$c$	2.328	2.503	1.881
		$0.5$	$m$	1.629	1.563	1.575
${\bar{\sigma }}_{{\theta \theta }_{max}}$	0.924	$1$	$m$	0.877	0.890	1.079
			$c$	0.953	0.978	0.972
		$0.5$	$m$	0.886	0.856	1.845

Overall, results show that there would be a possibility to reduce the stresses by higher percentages compared to the case presented in Table 3. It can be emphasized that there is a fluctuation in the stresses’ readings. However, at $\psi =90^\circ$ with ${\xi }_{{\beta }^{*}}=m$, ${\bar{\sigma }}_{{\theta \theta }_{max}}$ can be reduced by $\sim 7.3\%$ while ${\bar{\sigma }}_{{rr}_{max}}$ witnesses an insignificant increase ($<1\%$). This percentage of decline is greater by $\sim 2\%$ than the case where ${\beta }^{*}$ only included $\rho$. Thus, including both $\rho$ and $\mathbf{C}$ in Eq. (10) is more beneficial for the disc’s behaviors if compared to changing $\rho$ only, and the idea itself can produce encouraging outcomes (reducing failure/crack probability and raising the loading capacity). This showcases the originality of the proposed unconventional method.

On the other hand, it is not logical to investigate finite values of $\psi$ and ${x}_{{\beta }^{*}}$, with different material properties, loads, and geometry. Therefore, it can be stated that performing an adequate optimization for the current problem’s parameters is essential to produce the utmost stresses lessening levels, which is beyond the scope of the study.

### Case (2): Magneto-thermoelastic (MTE) loading

**Figure 6:**
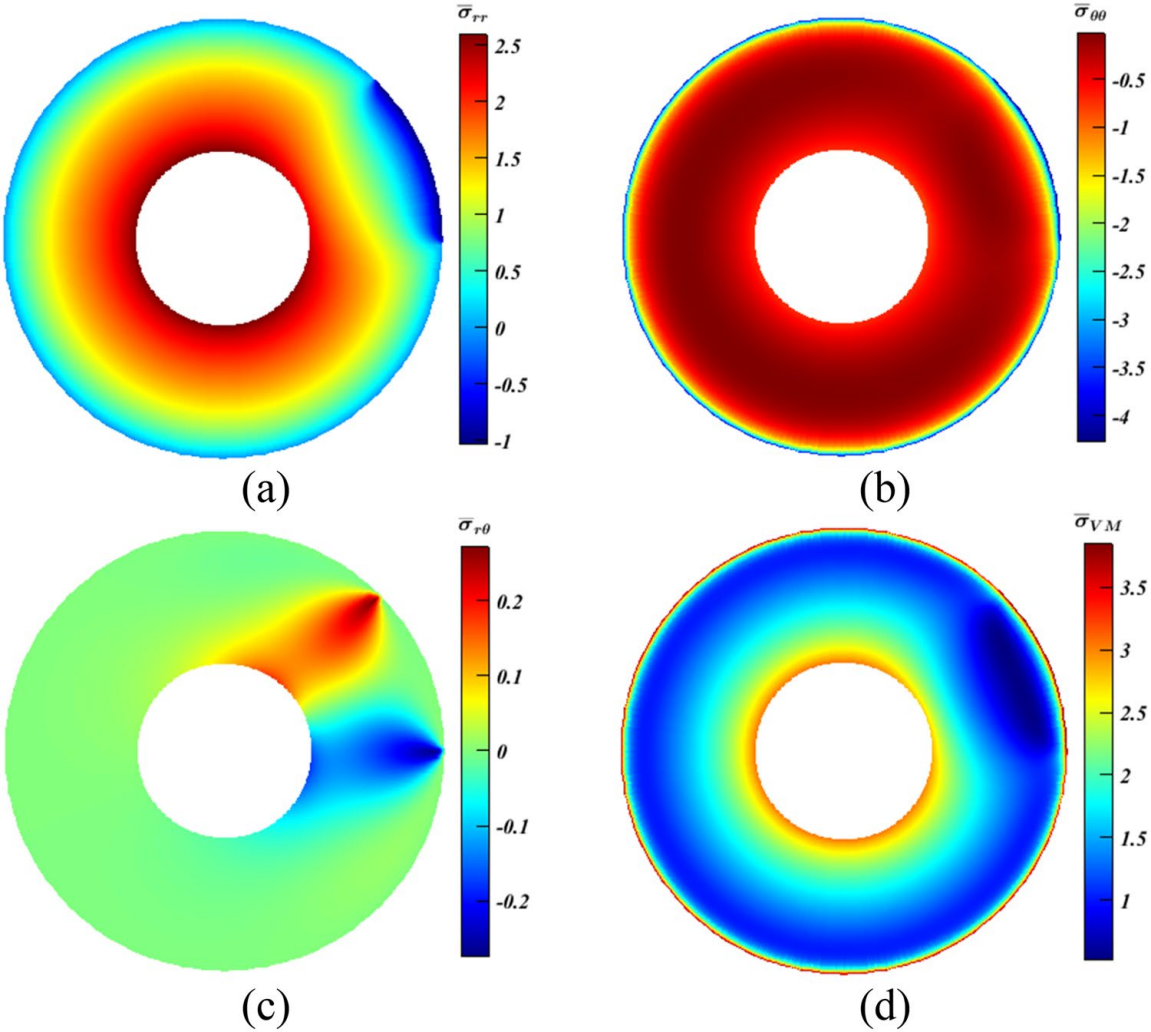
Dimensionless MTE stress contours for a rotating disc. (**a**) ${\bar{\sigma }}_{rr}$, (**b**) ${\bar{\sigma }}_{\theta \theta }$, (**c**) ${\bar{\sigma }}_{r\theta }$, and (**d**) ${\bar{\sigma }}_{\mathrm{vM}}$.

In this case, more complications are seen compared to the previous problem. This is achieved by considering the existence of thermal loading which resembles a more practical scenario. For that sake, the following BCs are used: $T({r}_{i},\theta )=100^\circ{\rm C}$, and $T({r}_{o},\theta )=500^\circ{\rm C}$ with the previous ones listed in “Case (1): Magnetoelastic (ME) loading” Section. As seen in Fig. 6, ${\bar{\sigma }}_{r{r}_{max}}$ stood at $2.6$, ${\bar{\sigma }}_{\theta {\theta }_{min}}=-4.289$, and ${\bar{\sigma }}_{r\theta }=\pm 0.27$. The last one would be neglected owing to its minor value^28^. The concentration would be directed towards the first two ones even though the absolute value of ${\bar{\sigma }}_{\theta {\theta }_{min}}$ is much larger than ${\bar{\sigma }}_{r{r}_{max}}$. Nonetheless, it can be said that both are important for ceramics that have tensile strength far less than the corresponding compressive strength. Thus, it can be stated that even if results yielded moderate growth for ${\bar{\sigma }}_{{\theta \theta }_{max}}$ and some dilatation for ${\bar{\sigma }}_{{rr}_{max}}$, this can be pointed out to as a positive point.

Also, in this case, ${\bar{\sigma }}_{\mathrm{VM}}$ (Fig. 6d) is included and discussed. It is calculated according to Eq. (20)^8^, and its dimensionless form is ${\bar{\sigma }}_{\mathrm{VM}}={\sigma }_{\mathrm{VM}}/{P}_{0}$. Here, ${\bar{\sigma }}_{{\mathrm{VM}}_{max}}$ peaked at $3.87.$ It should be noted that ${\bar{\sigma }}_{{\mathrm{VM}}_{max}}$ is excluded from the previous case study since the objective is to prove the adequacy of the idea.

20 $$ \sigma_{{{\text{VM}}}} = \left( {\sigma_{rr}^{2} - \sigma_{rr} \sigma_{\theta \theta } + \sigma_{\theta \theta }^{2} + 3\sigma_{r\theta }^{2} } \right)^{1/2} $$

Similar to the previous case, in order to apply the novel idea for stress reduction; Eq. (10) is used along with Eq. (9), where ${\beta }^{*}$ would include: (1) $\rho$, (2) $\rho$ and $\alpha$, and (3) $\rho$, $\alpha$ and $\mathbf{C}$. This is done in order to obtain the utmost applicable alleviations for the three stress values: ${\bar{\sigma }}_{r{r}_{max}}$, ${\bar{\sigma }}_{\theta {\theta }_{min}}$ and ${\bar{\sigma }}_{{\mathrm{VM}}_{max}}$.

**Figure 7:**
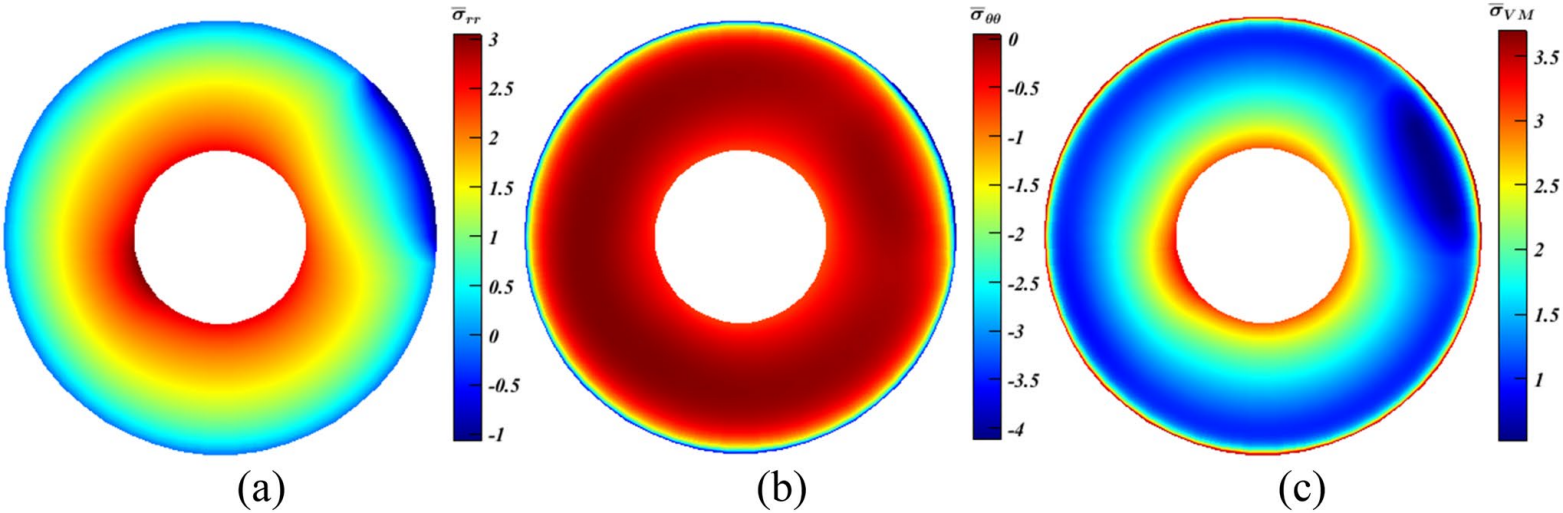
Dimensionless MTE stress contours for a rotating disc at $\psi =180^\circ$ with ${x}_{\rho }=1$ and ${\xi }_{\rho }=c$. (**a**) ${\bar{\sigma }}_{rr}$, (**b**) ${\bar{\sigma }}_{\theta \theta }$, and (**c**) ${\bar{\sigma }}_{\mathrm{VM}}$.

**Figure 8:**
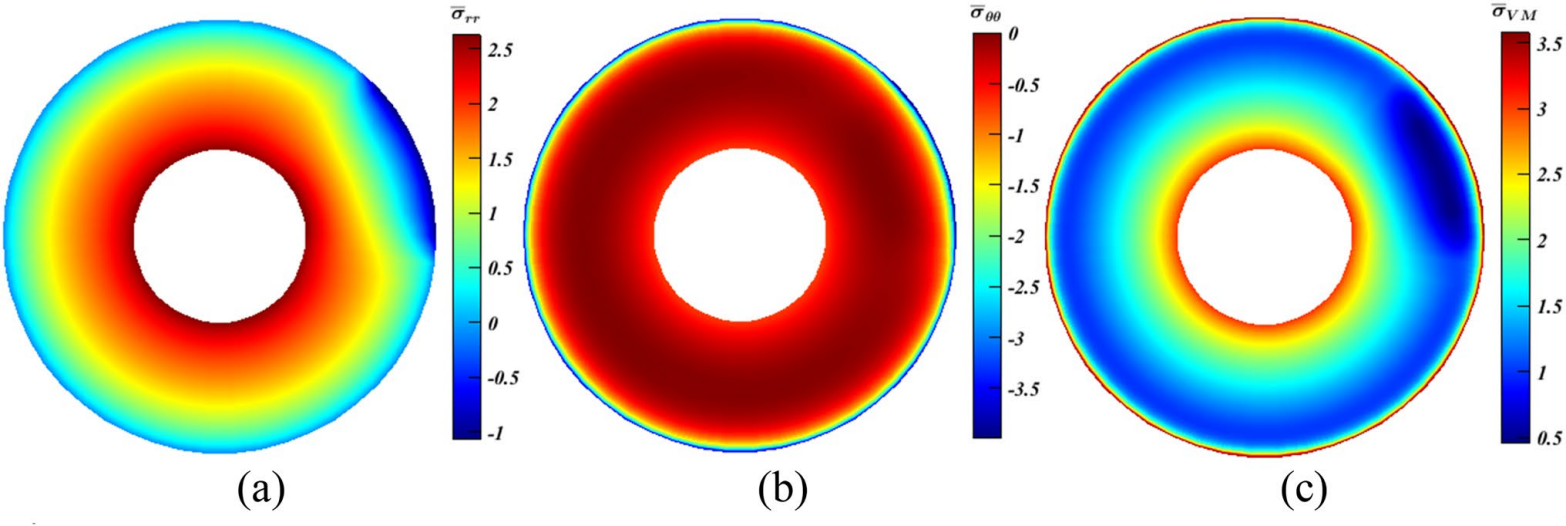
Dimensionless MTE stress contours for a rotating disc at $\psi =0^\circ$ with ${x}_{\rho }=1$ and ${\xi }_{\rho }=c$. (**a**) ${\bar{\sigma }}_{rr}$, (**b**) ${\bar{\sigma }}_{\theta \theta }$, and (**c**) ${\bar{\sigma }}_{\mathrm{VM}}$.

Starting with $\rho$ only in Eq. (10), a diverse variety of outcomes generated by altering $\psi$ and ${x}_{\rho }$, and for brevity, selected cases are debated ($\psi =180^\circ$ and $0^\circ$). At $\psi =180^\circ$ with ${x}_{\rho }=1$ and ${\xi }_{\rho }=c$, a tiny reduction of around $3\%$ occurred for ${\bar{\sigma }}_{\theta {\theta }_{min}}$ (Fig. 7b), whereas ${\bar{\sigma }}_{r{r}_{max}}$ grew by about $17\%$ according to Fig. 7a. Despite the fact that the increase is nearly six times the value of the decrease, ${\bar{\sigma }}_{{\mathrm{VM}}_{max}}$ is impacted moderately, as it declined by nearly $3.5\%$ as seen in Fig. 7c. This value experienced nearly a doubling hitting $6.2\%$ at $\psi =0^\circ$ with ${x}_{\rho }=1$ and ${\xi }_{\rho }=c$ (Fig. 8c), where ${\bar{\sigma }}_{\theta {\theta }_{min}}$ (Fig. 8b) dropped by nearly $5.1\%$ in contrast to ${\bar{\sigma }}_{r{r}_{max}}$ (Fig. 8a) that rose slightly by approximately $1.4\%$. On the contrary, results revealed that both ${\bar{\sigma }}_{r{r}_{max}}$ and ${\bar{\sigma }}_{\theta {\theta }_{min}}$ increased by < 1% and $8.5\%$, respectively, at similar $\psi$ while ${x}_{\rho }=0.5$ and ${\xi }_{\rho }=m$. Accordingly, ${\bar{\sigma }}_{{\mathrm{VM}}_{max}}$ jumped by $\sim 9\%$. Moreover, another interesting finding should be stated which is, while ${\beta }^{*}$ only included $\rho$, results at ${\xi }_{\rho }=c$ are better than that at ${\xi }_{\rho }=m$. This is traced back to ${\rho }_{c}>{\rho }_{m}$; hence ${F}_{r}$ becomes larger, and this works in opposite direction to the outer pressure especially at $\psi =0^\circ$.

Based on these selected results, a maximum feasible decline of ${\bar{\sigma }}_{{\mathrm{VM}}_{max}}$ is nearly $6.2\%$ while ${\beta }^{*}$ included $\rho$ only. Thus, the novel proposed method within the manuscript could play an essential role in alleviating the stresses that can in turns lead to a reduction in the failure likelihood and an increase in the loading limits. Moreover, seeking larger mitigation for the stresses; another trial is conducted through letting $\alpha$ join $\rho$ in Eq. (10). Table 5 lists the variation percentage detected for both ${\bar{\sigma }}_{{rr}_{max}}$ and ${\bar{\sigma }}_{{\theta \theta }_{min}}$ according to the authors’ records.

**Table 5:** Stresses variations’ percentages at different ${\varvec{\psi}}$ by varying ${\varvec{\rho}}$ and $\boldsymbol{\alpha }$ based on Eq. (10).

Stress	Reference value	Equation (10)		$\psi$		
		${x}_{\rho }={x}_{\alpha }$	${\xi }_{{\beta }^{*}}$	$180^\circ$	$90^\circ$	$0^\circ$
${\bar{\sigma }}_{{rr}_{max}}$	$2.6$	1	$m$	$0.98$	$1.104$	$0.873$
			$c$	$22.9$	$26.770$	$10.4$
		0.5	$m$	$0.547$	$0.196$	$0.548$
			$c$	$-0.22$	$5.25$	$-0.2$
${\bar{\sigma }}_{{\theta \theta }_{min}}$	$-4.289$	1	$m$	$114.2$	$116.453$	$128.5$
			$c$	$14.1$	$14.373$	$28.3$
		0.5	$m$	$42.3$	$42.506$	$56.6$
			$c$	$-0.97$	$5.9$	$14.83$

As seen, all the percentages are positive with different levels at ${x}_{{\beta }^{*}}=1$. Conversely, at ${x}_{{\beta }^{*}}=0.5$, ${\bar{\sigma }}_{{rr}_{max}}$ and ${\bar{\sigma }}_{{\theta \theta }_{min}}$ experienced some minor declines. Nevertheless, at the two values of ${x}_{{\beta }^{*}}$, ${\bar{\sigma }}_{{\mathrm{VM}}_{max}}$ experienced gigantic surges that are traced back to the interaction between the loads, ${\theta }_{P}$, material properties, and the geometry.

Another finding can be comprehended from Table 5 that is, at any $\psi$ and ${x}_{{\beta }^{*}}=0.5$, using ${\xi }_{{\beta }^{*}}=c$ yields smaller growths for ${\bar{\sigma }}_{\theta {\theta }_{min}}$ compared to using ${\xi }_{{\beta }^{*}}=m$. This concludes that using ${\xi }_{\alpha }=c$ would yield enhanced results compared to ${\xi }_{\alpha }=m$. In other words, the reduction of the prescribed area $\alpha$ is beneficial for the disc’s performance as it reduces the thermal strains, and accordingly, the thermal stresses. However, generally, it can be drawn that under the current conditions, permitting ${\beta }^{*}$ to include $\alpha$ along with $\rho$ has deleterious consequences on the disc’s performance.

**Figure 9:**
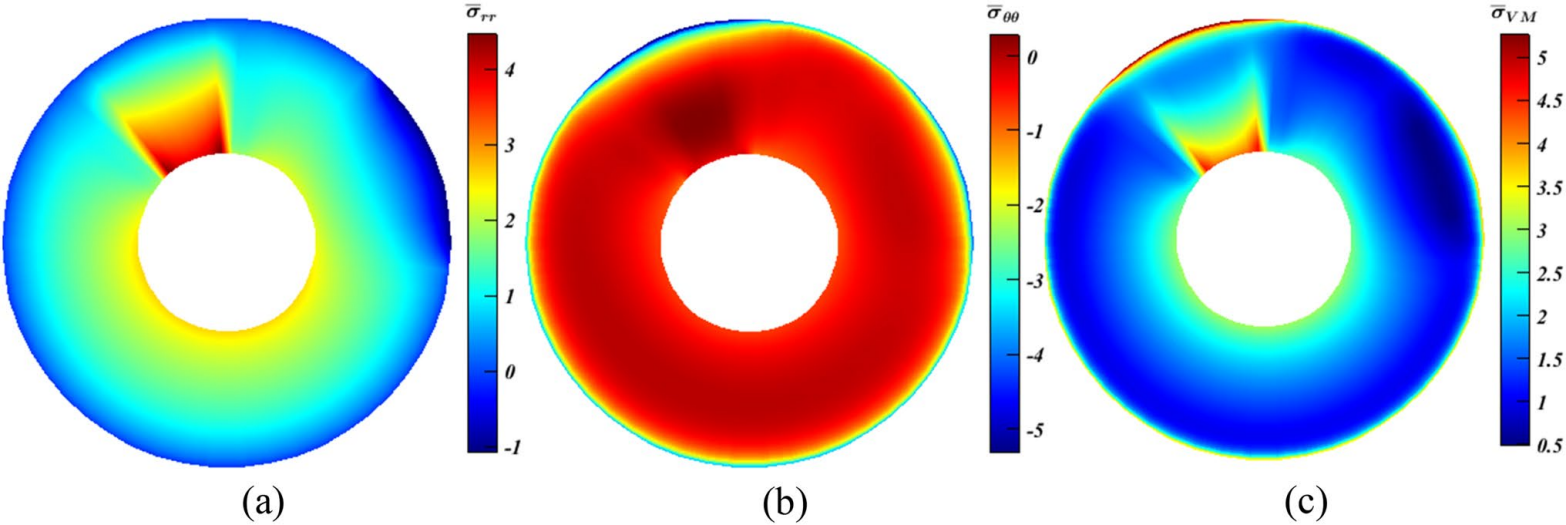
Dimensionless MTE stress contours for a rotating disc at $\psi =90^\circ$ with ${x}_{{\beta }^{*}}=1$ and ${\xi }_{{\beta }^{*}}=c$. (**a**) ${\bar{\sigma }}_{rr}$, (**b**) ${\bar{\sigma }}_{\theta \theta }$, and (**c**) ${\bar{\sigma }}_{\mathrm{VM}}$. ${\beta }^{*}$ includes $\mathrm{C}$, $\rho$ and $\alpha$.

Afterwards, as done in Case (1), Eq. (10) is extended to $\mathbf{C}$ as well as $\rho$ and $\alpha$ so as to try achieving higher stress alleviation levels. Unfortunately, records disclose that both ${\bar{\sigma }}_{{rr}_{max}}$ and ${\bar{\sigma }}_{{\theta \theta }_{min}}$ increase substantially by using any value of $\psi$ at ${x}_{{\beta }^{*}}=1$. Hence, ${\bar{\sigma }}_{{\mathrm{VM}}_{max}}$ goes up as well. For instance, at $\psi =90^\circ$ with ${x}_{{\beta }^{*}}=1$ and ${\xi }_{{\beta }^{*}}=c$ (Fig. 9), ${\bar{\sigma }}_{{rr}_{max}}$, ${\bar{\sigma }}_{{\theta \theta }_{min}}$ and ${\bar{\sigma }}_{{\mathrm{VM}}_{max}}$ significantly soared by $87\%$, $23\%$ and $36\%$, respectively.

In contrast, using ${x}_{\beta }=0.5$ produced enhanced reductions for the three stresses as outlined in Table 6. It is seen that there are advantageous falls in the values of ${\bar{\sigma }}_{{rr}_{max}}$ and ${\bar{\sigma }}_{{\theta \theta }_{min}}$ leading to noteworthy plunges of ${\bar{\sigma }}_{\mathrm{VM}}$ regardless of $\psi$. For example, at $\psi =0^\circ$, ${\bar{\sigma }}_{{\mathrm{VM}}_{max}}$ dropped by approximately $8.9\%$ and $9.9\%$ at ${\xi }_{{\beta }^{*}}=m$ and $c$, respectively, and these two values surpass the applicable reductions levels at $\psi =90^\circ$ and $180^\circ$. These results again prove that there would be stresses’ reductions and an increase for the loading capacity through using the method proposed within this study.

**Table 6:** Stress variation percentage at different ${\varvec{\psi}}$ by varying ${\varvec{\rho}}$, $\boldsymbol{\alpha }$ and $\mathbf{C}$ according to Eq. (10) using ${{\varvec{x}}}_{{{\varvec{\beta}}}^{\boldsymbol{*}}}=0.5$.

Stress	Reference value	$\xi_{{\beta^{*} }}$	$\psi$		
			$180^\circ$	$90^\circ$	$0^\circ$
$\overline{\sigma }_{{rr_{max} }}$	$2.6$.	$m$	$- 0.457$	$- 0.367$	$- 0.158$
		$c$	$- 0.392$	$1.976$	$- 0.340$
$\overline{\sigma }_{{\theta \theta_{min} }}$	$- 4.289$	$m$	$- 1.352$	$- 2.578$	$- 17.2$
		$c$	$- 1.923$	$- 3.630$	$- 18.109$
$\overline{\sigma }_{{{\text{VM}}_{\max } }}$	$3.87$	$m$	$-1.456$	$-2.775$	$-8.97$
		$c$	$-2.070$	$-3.906$	$-9.9$

Finally, and opposing to the previous results where ${x}_{{\beta }^{*}}$ has an equal value for the three properties; having different configurations of it are examined at only $\psi =0^\circ$. For that sake, the following values were used: ${\xi }_{\rho }={\xi }_{\alpha }=c$, ${\xi }_{\mathbf{C}}=m$, ${x}_{\rho }=1$, ${x}_{\alpha }=0.25$, and ${x}_{\mathbf{C}}\hspace{0.17em}=\hspace{0.17em}$1, $0.5$ or $0.25$.

**Figure 10:**
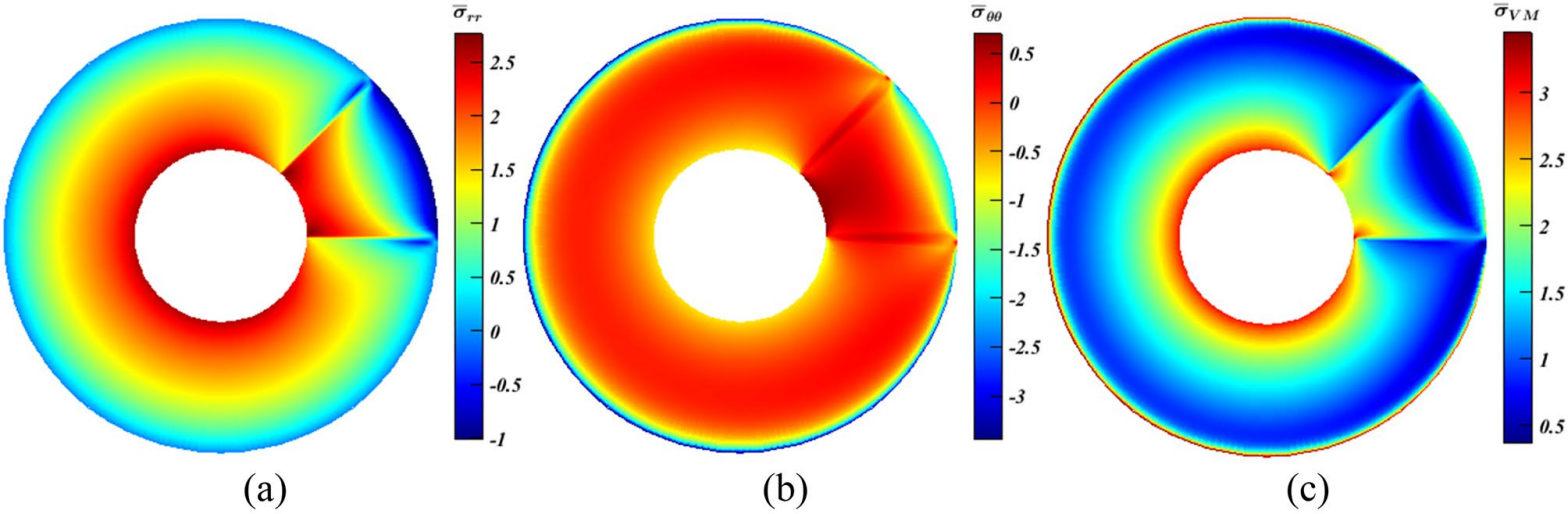
Dimensionless MTE stress contours for a rotating disc at $\psi =0^\circ$ with ${x}_{\rho }={x}_{\mathbf{C}}=4{x}_{\alpha }=1$, ${\xi }_{\rho }={\xi }_{\alpha }=c$ and ${\xi }_{\mathbf{C}}=m$. (**a**) ${\bar{\sigma }}_{rr}$, (**b**) ${\bar{\sigma }}_{\theta \theta }$, and (**c**) ${\bar{\sigma }}_{\mathrm{VM}}$.

**Figure 11:**
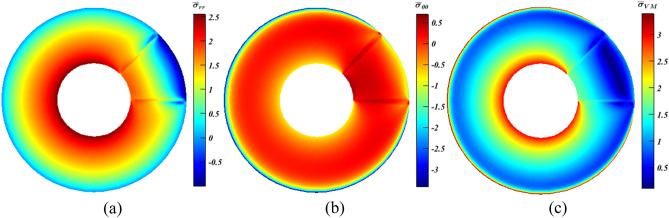
Dimensionless MTE stress contours for a rotating disc at $\psi =0^\circ$ with ${x}_{\rho }=2{x}_{\mathbf{C}}=4{x}_{\alpha }=1$, ${\xi }_{\rho }={\xi }_{\alpha }=c$ and ${\xi }_{\mathbf{C}}=m$. (**a**) ${\bar{\sigma }}_{rr}$, (**b**) ${\bar{\sigma }}_{\theta \theta }$, and (**c**) ${\bar{\sigma }}_{\mathrm{VM}}$.

**Figure 12:**
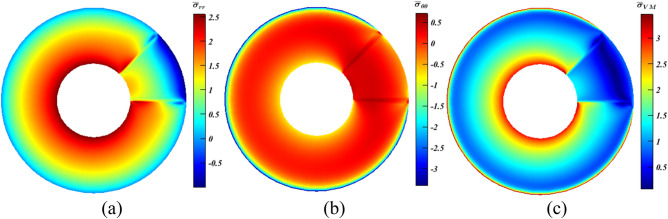
Dimensionless MTE stress contours for a rotating disc at $\psi =0^\circ$ with ${x}_{\rho }=4{x}_{\mathbf{C}}=4{x}_{\alpha }=1$, ${\xi }_{\rho }={\xi }_{\alpha }=c$ and ${\xi }_{\mathbf{C}}=m$. (**a**) ${\bar{\sigma }}_{rr}$, (**b**) ${\bar{\sigma }}_{\theta \theta }$, and (**c**) ${\bar{\sigma }}_{\mathrm{VM}}$.

As portrayed in Fig. 10a, ${\bar{\sigma }}_{{rr}_{max}}$ witnessed substantial increase ($\sim 8\%$), while in Figs. 11a and 12a, it experienced slight declines ($<2\%$). Conversely, ${\bar{\sigma }}_{{\theta \theta }_{min}}$ fell by approximately $19.8\%$ at ${x}_{\mathbf{C}}=1$ (Fig. 10b), and continued to decrease reaching more or less than $20.7\%$, which can be considered as a great achievement, at ${x}_{\mathbf{C}}=0.5$ and $0.25$ as seen in Figs. 11b and 12b, respectively. Thus, according to the readings of ${\bar{\sigma }}_{{rr}_{max}}$ and ${\bar{\sigma }}_{{\theta \theta }_{min}}$, the smaller the value of ${x}_{\mathbf{C}}$, the higher the stresses’ reductions are attainable. However, this is not dominant for the values of $\overline{\sigma }_{{{\text{VM}}_{max} }}$. At $x_{{\mathbf{C}}} = 1$ (Fig. 10c), it dropped by a large value ($\sim 10.8\%$), and continued to declined hitting nearly $12.5\%$ at $x_{{\mathbf{C}}} = 0.5$ (Fig. 11c). As $x_{{\mathbf{C}}}$ shrinks to $0.25$, a lessening of $10.5\%$ is experienced as seen in Fig. 12c.

These results prove the valuable impacts of the idea of keeping some the properties constant through a selected area within the disc to reduce the failure likelihood and/or raise the working load capacity and safety. They also confirm that the problem in hand is nonlinear; thus, the behaviors are hardly predicted. Accordingly, performing an adequate optimization is central to obtain the most suitable values of ${x}_{{\beta }^{*}}$ and $\psi$. Additionally, it opens new horizons through considering more properties within the prescribed area that can have different values of $\psi$ other the one used within the current study.

## Conclusion

The main contribution of this article is trying to open new horizons for lowering the magnetoelastic/magneto-thermoelastic stresses and/or increasing the working limits of rotating FGM discs. This was sought through an atypical idea that presumes the existence of an area within the disc with definite dimensions, location, and particular homogeneous properties. The assumption of perfect bonding between this area and the FGM area was utilized in the analyses. The solution of the governing equations was performed through FEM.

Overall, results showed that some constructive stress reductions were achievable depending on the location and the chosen constant properties of the prescribed area. A decline of $\sim 7.3\%$ in the maximum tensile tangential stress occurred for the ME loading case. This percentage jumped to nearly $20.7\%$ at the MTE case, which led to mitigation of the von Mises stress by about $12.5\%$ at certain composition for the homogenous area.

These results were indisputably beneficial for decreasing the crack formation/propagation likelihood and/or raising the payload limits, which proves the originality of the proposed method. However, optimizing many parameters is recommended to obtain the utmost stresses suppression especially for such nonlinear problems. These parameters include, for instance, thickness, property and its fraction, location, and the dimension of the homogenous area.

## Data Availability

The datasets generated and/or analyzed during the current study are not publicly available due to being part of an ongoing study but are available from the first or the third authors on a reasonable request.
